# Screening of Polyvalent Phage-Resistant *Escherichia coli* Strains Based on Phage Receptor Analysis

**DOI:** 10.3389/fmicb.2019.00850

**Published:** 2019-04-18

**Authors:** Ping Li, Hong Lin, Zhiqiang Mi, Shaozhen Xing, Yigang Tong, Jingxue Wang

**Affiliations:** ^1^Food Safety Laboratory, Department of Food Science and Engineering, Ocean University of China, Qingdao, China; ^2^State Key Laboratory of Pathogens and Biosecurity, Beijing Institute of Microbiology and Epidemiology, Beijing, China; ^3^Department of Life Science and Technology, Beijing University of Chemical Technology, Beijing, China

**Keywords:** *Escherichia coli* BL21 (DE3), phage contamination, phage-host interaction, phage resistance, phage receptors

## Abstract

Bacteria-based biotechnology processes are constantly under threat from bacteriophage infection, with phage contamination being a non-neglectable problem for microbial fermentation. The essence of this problem is the complex co-evolutionary relationship between phages and bacteria. The development of phage control strategies requires further knowledge about phage-host interactions, while the widespread use of *Escherichia coli* strain BL21 (DE3) in biotechnological processes makes the study of phage receptors in this strain particularly important. Here, eight phages infecting *E. coli* BL21 (DE3) via different receptors were isolated and subsequently identified as members of the genera *T4virus*, *Js98virus*, *Felix01virus*, *T1virus*, and *Rtpvirus*. Phage receptors were identified by whole-genome sequencing of phage-resistant *E. coli* strains and sequence comparison with wild-type BL21 (DE3). Results showed that the receptors for the isolated phages, designated vB_EcoS_IME18, vB_EcoS_IME253, vB_EcoM_IME281, vB_EcoM_IME338, vB_EcoM_IME339, vB_EcoM_IME340, vB_EcoM_IME341, and vB_EcoS_IME347 were FhuA, FepA, OmpF, lipopolysaccharide, Tsx, OmpA, FadL, and YncD, respectively. A polyvalent phage-resistant BL21 (DE3)-derived strain, designated PR8, was then identified by screening with a phage cocktail consisting of the eight phages. Strain PR8 is resistant to 23 of 32 tested phages including *Myoviridae* and *Siphoviridae* phages. Strains BL21 (DE3) and PR8 showed similar expression levels of enhanced green fluorescent protein. Thus, PR8 may be used as a phage resistant strain for fermentation processes. The findings of this study contribute significantly to our knowledge of phage-host interactions and may help prevent phage contamination in fermentation.

## Introduction

Bacteriophage, which were first discovered by Twort in 1915 and confirmed by d’Hérelle in 1917, are a highly diverse group of viruses that are ten times more abundant than their bacterial hosts in most environments (Brussow and Hendrix, 2002; Paez-Espino et al., 2016). As alternative antimicrobials, phages have been used to achieve effective bacterial control in various food, aquaculture, clinical, biotechnology, and other industries, especially for the treatment of drug-resistant strains (Salmond and Fineran, 2015). However, phage contamination is a non-neglectable problem for biotechnology- and food industry-based microbial fermentation processes, where the resulting losses can be catastrophic (Ogata, 1980; Marco et al., 2012). To address this, several effective phage monitoring systems and control measures have been developed and implemented in recent years, including control of contamination sources, rotation of different phage-resistant strains, genetic engineering strategies targeting different stages of phage infection, and phage control strategies based on the clustered regularly interspaced short palindromic repeats (CRISPR)-Cas system (Sturino and Klaenhammer, 2006; Marco et al., 2012; Samson and Moineau, 2013).

There is a continuous co-evolutionary relationship between phages and their hosts, in which phage-resistant bacterial strains help preserve the bacterial lineage, with novel counter-resistant phages then threatening these strains (Labrie et al., 2010). Phage propagation involves several stages, including initial phage attachment to the surface of the host cell, followed by injection of phage nucleic acid into the host. The phage components are then synthesized intracellularly and packaged into self-assembling viral particles. Finally, an enzyme capable of degrading the bacterial cell wall is expressed, the bacteria are lysed, and the phage is released. Bacteria have evolved different phage resistance mechanisms, including prevention of phage adsorption, prevention of phage DNA entry, restriction/modification of phage nucleic acids, abortive infection systems and CRISPR-Cas systems (Hyman and Abedon, 2010; Labrie et al., 2010). Preventing phage adsorption is the first step in bacterial defense against phage, and includes mutations in phage receptors and production of extracellular matrices and competitive inhibitors (Hyman and Abedon, 2010; Labrie et al., 2010). Mutations in phage receptors are the most common way to prevent phage adsorption. Phage receptors located on the cell outer membrane of Gram-negative bacteria are mainly outer membrane proteins (OMPs) and lipopolysaccharides (LPS), but can sometimes be flagellum, pilus, or capsular proteins (Bertozzi Silva et al., 2016). For example, OmpC is the receptor for both *Myoviridae* phage Me1 and *Siphoviridae* phage Gifsy-1 (Verhoef et al., 1977; Ho and Slauch, 2001), while the receptor for *Myoviridae* phages M1 and Ox2 is OmpA (Morona and Henning, 1984; Hashemolhosseini et al., 1994). Similarly, the receptor for *Siphoviridae* phages BF23 and SPN7C is outer membrane transport protein BtuB (Di et al., 1973; Shin et al., 2012), while LPS is recognized as the receptor for *Podoviridae* phage T3 and *Myoviridae* phage JG004 (Prehm et al., 1976; Boyke et al., 2011). The receptor for *Siphoviridae* phage iEPS5 is the flagellar molecular ruler protein FliK (Choi et al., 2012), while the receptor for *Siphoviridae* phage MP22 and *Podoviridae* phage MPK7 is the Type IV pilus (Heo et al., 2007; Bae and Cho, 2013). The bacterial capsule has been identified as the receptor for *Myoviridae* phage Vi I, *Siphoviridae* phage Vi II, and *Podoviridae* phage Vi III (Pickard et al., 2010). Interestingly, most *Siphoviridae* phages infecting Gram-negative bacteria require protein receptors for adsorption, while most *Podoviridae* phages require polysaccharides for the same process (Bertozzi Silva et al., 2016). Identification of phage receptors is the first step in studying phage-host interactions.

*Escherichia coli* is the most commonly used species in recombinant protein expression systems because such systems are rapid, simple, inexpensive, and allow large-scale production of target proteins. *E. coli* strain BL21 (DE3) was specifically designed for the overexpression of recombinant proteins. Understanding phage-host interactions, particularly the phage receptors of strain BL21 (DE3), is essential for the development of next-generation anti-phage strategies (Mahony and van Sinderen, 2015). However, the receptors of strain BL21 (DE3) have not been extensively studied. In previous work, we reported partial information about, and receptors for, phage vB_EcoS_IME347 (Li et al., 2018). Here, phages that recognize different receptors on BL21 (DE3) were sequentially screened and then combined in a “phage cocktail” to identify a polyvalent phage-resistant *E. coli* strain derived from BL21 (DE3). We confirmed that FhuA, FepA, OmpF, LPS, Tsx, OmpA, FadL, and YncD can be used as receptors by BL21 (DE3)-infecting phages. Together, our findings provide data support for phage-host interaction studies and will aid in the control of phage contamination of *E. coli* BL21 (DE3).

## Materials and Methods

### Bacterial Strains, Plasmids, and Culture Conditions

Bacteria and plasmids used in this study are listed in Table 1. All primers used are listed in Supplementary Table S1. The Sanger sequencing used in this study was completed by Beijing Tianyi Huiyuan Biotechnology Co., Ltd. *E. coli* strain BL21 (DE3; GenBank Accession No. CP001509) was stored at −70°C in 25% (v/v) glycerol. Plasmids pKDsg-ack, pCas9cr4, and pET-28a were stored at −20°C. All bacterial strains were cultured in Luria-Bertani (LB) broth (tryptone, 10 g/L; yeast extract, 5 g/L; NaCl, 5 g/L). Medium was supplemented with agar (1.5%), soft agar (0.75%), 100 mg/L ampicillin (Amp), 50 mg/L spectinomycin (Spec), 50 mg/L kanamycin (Kana), or 100 μg/L anhydrotetracycline (aTc) when necessary.

**Table 1:** Bacteria and plasmids used in this study.

Bacteria and plasmids	Description	
Bacteria	Description	Source
*Escherichia coli* BL21 (DE3)	Phage host strain	TransGen company
18-R (18-R1, 18-R2, 18-R3)	Resistant mutant strains of phage vB_EcoS_IME18	This study
253-R (253-R1, 253-R2, 253-R3)	Resistant mutant strains of phage vB_EcoS_IME253	This study
281-R (281-R1, 281-R2, 281-R3)	Resistant mutant strains of phage vB_EcoM_IME281	This study
338-R (338-R1, 338-R2, 338-R3)	Resistant mutant strains of phage vB_EcoM_IME338	This study
339-R (339-R1, 339-R2, 339-R3)	Resistant mutant strains of phage vB_EcoM_IME339	This study
340-R (340-R1, 340-R2, 340-R3)	Resistant mutant strains of phage vB_EcoM_IME340	This study
341-R (341-R1, 341-R2, 341-R3)	Resistant mutant strains of phage vB_EcoM_IME341	This study
347-R (347-R1, 347-R2, 347-R3)	Resistant mutant strains of phage vB_EcoS_IME347	This study
Δ*tonB*	Deletion mutants of *tonB*	This study
Δ*fhuA*	Deletion mutants of *fhuA*	This study
Δ*fepA*	Deletion mutants of *fepA*	This study
Δ*ompF*	Deletion mutants of *ompF*	This study
Δ*waaG*	Deletion mutants of *waaG*	This study
Δ*tsx*	Deletion mutants of *tsx*	This study
Δ*ompA*	Deletion mutants of *ompA*	This study
Δ*fadL*	Deletion mutants of *fadL*	This study
Δ*yncD*	Deletion mutants of *yncD*	This study
*C-tonB*	Complementary strains of *tonB*	This study
*C-fhuA*	Complementary strains of *fhuA*	This study
*C-fepA*	Complementary strains of *fepA*	This study
*C-ompF*	Complementary strains of *ompF*	This study
*C-waaG*	Complementary strains of *waaG*	This study
*C-tsx*	Complementary strains of *tsx*	This study
*C-ompA*	Complementary strains of *ompA*	This study
*C-fadL*	Complementary strains of *fadL*	This study
*C*-*yncD*	Complementary strains of *yncD*	This study
Plasmids	Description	Source
pCas9cr4	For scarless Cas9 assisted recombineering (no-SCAR) system	Addgene (Plasmid #62655)
pKDsg-ack	For scarless Cas9 assisted recombineering (no-SCAR) system	Addgene (Plasmid #62654)
pKDsg-p15	For scarless Cas9 assisted recombineering (no-SCAR) system	Addgene (Plasmid #62656)
pKDsg-tonB	Counter-selection plasmid for deletion of *tonB*	This study
pKDsg-fhuA	Counter-selection plasmid for deletion of *fhuA*	This study
pKDsg-fepA	Counter-selection plasmid for deletion of *fepA*	This study
pKDsg-ompF	Counter-selection plasmid for deletion of *ompF*	This study
pKDsg-waaG	Counter-selection plasmid for deletion of *waaG*	This study
pKDsg-tsx	Counter-selection plasmid for deletion of *tsx*	This study
pKDsg-ompA	Counter-selection plasmid for deletion of *ompA*	This study
pKDsg-fadL	Counter-selection plasmid for deletion of *fadL*	This study
pET-28a-tonB	Complementation plasmids for complementation of *tonB*	This study
pET-28a-fhuA	Complementation plasmids for complementation of *fhuA*	This study
pET-28a-fepA	Complementation plasmids for complementation of *fepA*	This study
pET-28a-ompF	Complementation plasmids for complementation of *ompF*	This study
pET-28a-waaG	Complementation plasmids for complementation of *waaG*	This study
pET-28a-tsx	Complementation plasmids for complementation of *tsx*	This study
pET-28a-ompA	Complementation plasmids for complementation of *ompA*	This study
pET-28a-fadL	Complementation plasmids for complementation of *fadL*	This study
pET-28a-egfp	Recombinant plasmid to identify differences in protein expression between polyvalent phage-resistant strain PR8 and *E. coli* BL21 (DE3)	This study

### Isolation and Purification of Phages and Screening of Bacteriophage-Insensitive Mutants

Phages specific to *E. coli* strain BL21 (DE3) were isolated from sewage samples collected from the State Key Laboratory of Pathogens and Biosecurity, Beijing Institute of Microbiology and Epidemiology, Beijing, China. The sewage samples were centrifuged at 13,000 × *g* for 10 min and then filtered through a 0.22-μm pore size filter (Millipore, Burlington, MA, United States). Thereafter, 4 mL of the filtrate were mixed with 200 μL of log-phase BL21 (DE3) cells [optical density at 600 nm (OD_600_) = 0.6] and inoculated into 2 mL of 3 × LB medium before being incubated at 37°C, 220 rpm until clarification. The resulting culture suspension was filtered as above and the phages were cultured using the double-layer agar plate method as described previously (Jun et al., 2016). An individual plaque was picked for purification from three separate replicates. The purified phage particles were stored in LB medium containing 25% (v/v) glycerol at −80°C.

Log-phase BL21 (DE3) suspension (OD_600_ = 0.6, 300 μL) and phage suspensions (10^6^ plaque-forming units [PFU]/mL, 100 μL) obtained in the above procedure were mixed with soft agar and then poured onto the surface of LB-agar plates. The plates were incubated at 37°C for 12–18 h or until colonies were produced on the plaques. Single bacterial colonies were obtained by streaking, and their sensitivity to phage infection was examined using a spotting assay and a double-layer agar plate method (McCutcheon et al., 2018). A single colony from plates on which no plaques were formed was selected as a potential bacteriophage-insensitive mutant (BIM).

Each phage and its BIMs was screened using an iterative mutagenesis method using a previous generation of BIMs as an indicator strain to screen the next generation of phage, and then screening for new BIMs (Supplementary Figure S1).

### Transmission Electron Microscopy (TEM)

Phage particles were centrifuged at 13,000 × *g* for 1h then purified by CsCl gradient ultra-centrifugation to visualize phage morphology by TEM (Chen et al., 2018). A 20-μL aliquot of phage suspension was incubated on a carbon-coated copper grid for 15 min and then dried using filter paper. The copper grid covering the phages was then stained with 2% (w/v) phosphotungstic acid for 2 min. Finally, phage morphology was examined at 80 kV using a JEM-1200EX transmission electron microscope (Jeol Ltd., Tokyo, Japan).

### Optimal Multiplicity of Infection (MOI) and One-Step Growth Curve Analyses

The optimal MOI and one-step growth curves for isolated phages were determined using methods described previously (Wang et al., 2014). Briefly, phages were added to 5 mL of log-phase BL21 (DE3) culture (10^8^ colony-forming units [CFU]/mL) to achieve a MOI of 10, 1, 0.1, 0.01, 0.001, or 0.0001, and then incubated at 37°C, 220 rpm for 4 h. Culture supernatant was then filtered through a 0.22-μm filter, and the titer of the phage in the supernatant was measured using a double-layer agar plate method. Three replicates were conducted for determination. The MOI resulting in the highest phage titer was considered the optimal MOI of the phage.

The one-step growth curve of a phage reflects dynamic changes in the number of particles during phage replication. To obtain a one-step growth curve for each of the isolated phages, phage suspension was added to 20 mL of log-phase BL21 (DE3) culture (10^7^ CFU/mL) at the optimal MOI and incubated at 37°C for 5 min. The culture was then centrifuged at 12,000 × *g* for 1 min and the supernatant discarded. The pellet was then washed twice with LB medium and re-suspended in 20 mL of LB medium. The moment when the pellet was re-suspended in medium was defined as time zero. Then, the resulting culture was transferred to a shaker and incubated at 37°C, 220 rpm for 1.5 h. Three duplicate samples (200 μL) were collected every 10 min to determine the phage titer at different time points. Three replicates were conducted for determination. The one-step growth curve was obtained by plotting phage titer against time. The burst size was calculated by dividing the plateau phage titer by the initial phage titer.

### Gene Sequencing and Bioinformatic Analysis

Phage genomic DNA was extracted using a modified phenol-chloroform extraction protocol (Zhang et al., 2017). A 2 × 300 nt paired-end DNA library was prepared with the NEBNext^®^ Ultra^TM^ II DNA Library Prep Kit for Illumina following the manufacturer’s protocol. Briefly, 150 ng of DNA were dissolved in deionized water to a final volume of 50 μl and disrupted to 300–400 bp fragments using a Bioruptor UCD-200TS ultrasound system. Then, the fragmented DNAs were end-repaired and adaptor ligated using NEBNext Ultra II End Prep Enzyme and Ligation Master Mix, respectively. Next, the adaptor-ligated DNA was selected and cleaned using EBNext Sample Purification Beads. Finally, the adaptor-ligated DNA was subjected to PCR amplification, and the PCR products were cleaned using EBNext Sample Purification Beads. Before sequencing, quality-control analysis for the constructed library was performed for fragment size distribution with a Bioanalyzer 2100 (Agilent Technologies). Then, high-throughput sequencing of the DNA was performed on an Illumina MiSeq instrument (San Diego, CA, United States). Genomes were assembled from filtered high-quality reads using the assembly algorithm Newbler version 3.0 with default parameters. Open reading frame prediction and genome annotation were carried out using the RAST^1^ tool and NCBI nucleotide collection (non-redundant nr database) BLASTp alignment, respectively. A phylogenetic tree was constructed using 75 terminase large subunit protein sequences of different phages from the *Ounavirinae*, *Tevenvirinae*, and *Tunavirinae* subfamilies in the International Committee on Taxonomy of Viruses virus taxonomy current release. The alignment of phage terminase large subunit sequences was carried out using ClustalW in MEGA 6 software, and then phylogenetic analysis was performed using the maximum likelihood method based on the JTT matrix-based model with 1000 bootstrap replicates.

Genomic DNA was extracted from phage-resistant *E. coli* strains and phage-sensitive strain BL21 (DE3) using a High Pure PCR Template Preparation Kit (Roche, Mannheim, Germany). Then, a 2 × 300 nt paired-end DNA library was constructed using the Illumina NEB Next^®^ Ultra^TM^ II DNA library preparation kit and high-throughput sequencing was performed using Illumina MiSeq according to the manufacturer’s instructions. Sequence reads were mapped against the BL21 (DE3) reference genome (GenBank Accession No. CP001509.3) using CLC Genomics Workbench 9.0, followed by basic variant detection and gene deletion searches.

### Phage Sensitivity Assay

The sensitivity of bacterial strains to phages was examined by the double-layer agar plate method and adsorption assays. Phage-sensitive strains were selected to determine their phage adsorption capacity. Briefly, log-phase (10^8^ CFU/mL) bacterial culture (1 mL) was centrifuged at 12,000 × *g* for 1 min and the pellet resuspended in phosphate-buffered saline (0.9 mL). A 0.1 mL aliquot of phage solution (∼10^6^ PFU/mL) was added to the cell suspension and the culture mixture was incubated at 37°C for 5 min to allow adsorption. In controls, LB medium was used instead of a cell suspension. Cultures were then centrifuged at 12,000 × *g* for 1 min and the titer of free phage in the supernatant was determined using the double-layer agar method. Three replicates were conducted for determination. The adsorption rate is estimated by *k* = (*C_t_* − *P_t_*)/*P*_0_/*N*/*t*, where *k* is the estimated adsorption rate; *P_t_* and *P*_0_ is the phage concentration at time *t* and 0, respectively; *C_t_* is the phage concentration in control group at the time *t*; *N* is the bacterial concentration.

### Construction of Deletion Mutants

*Escherichia coli* BL21 (DE3) *tonB*, *fhuA*, *fepA*, *ompF*, *waaG*, *tsx*, *ompA*, and *fadL* deletion mutants were constructed using the Scarless Cas9 Assisted Recombineering (no-SCAR) system (Reisch and Prather, 2015, 2017). The no-SCAR system consists of plasmids pKDsg-ack and pCas9cr4, which contain all components required for gene editing and do not require specific modification of the host. pCas9cr4 expresses Cas9 nuclease under the control of the *P_tet_* promoter, while pKDsg-xxx has a single guide RNA (sgRNA) expressed under the control of *P_tet_* as well as *exo*, *bet*, and *gam* genes, which constitute the λ-Red system, under the control of the arabinose-inducible promoter, *P_araB_* (Reisch and Prather, 2015). Cas9 target recognition requires a protospacer adjacent motif (PAM, 5′-NGG-3′) next to the target site. Cas9 nuclease produces double-strand breaks in the genome at the target site under the guidance of the sgRNA, while the λ-Red system links the donor nucleic acid to the cut genome for gene editing.

The counter-selection plasmid pKDsg-xxx was reconstructed for Cas9 target specificity. The oligonucleotides used for recombination were designed as single-stranded DNA, and three phosphorothioate bonds were 5′-modified to prevent degradation. Briefly, the sgRNA sequence on plasmid pKDsg-xxx was designed using the online CRISPR gRNA design tool^2^ or by manual selection of 20-bp fragments of DNA at the 3′- end of the target gene PAM region. To incorporate this 20-bp sequence into pKDsg-xxx, a ligation-independent cloning technique known as circular polymerase extension cloning (CPEC) was performed (Quan and Tian, 2009). Amplification of two products with short, overlapping sequences on both ends was performed using Q5 HotStart High-Fidelity 2 × Master Mix (New England Biolabs, Ipswich, MA, United States) using pKDsg-ack as the template. The two linear DNA fragments were then assembled into plasmids using a pEASY-UniSeamless cloning and assembly kit (TransGen, Beijing, China) and transformed into Trans5α Chemically Competent Cells (TransGen). Transformed cells were incubated on LB agar containing 50 mg/L Spec for 12 h at 30°C. The presence of pKDsg-xxx in the transformants was confirmed by PCR. Plasmids were then extracted using a Plasmid Mini Kit (TaKaRa, Otsu, Japan).

Deletion mutant strains were then constructed as follows. First, plasmids pCas9cr4 and pKDsg-xxx were sequentially transformed into *E. coli* BL21 (DE3) by electroporation and selected using Amp and Spec, respectively. Transformed plasmids were verified by PCR. Next, BL21 (DE3) transformants containing pCas9cr4 and pKDsg-xxx were cultured to log phase for preparation of competent cells. Cells were incubated with 50 mM (final concentration) L-arabinose for 20–30 min before preparation of competent cells to induce expression of the λ-Red recombination system in pKDsg-xxx. Finally, the target oligonucleotide was electro transformed into the recombinant competent cells, which were then plated on LB-agar containing 100 mg/L Amp, 50 mg/L Spec, and 100 μg/L aTc, and incubated at 30°C for 12 h.

### Genotyping

Single colonies of deletion mutant strains were genotyped by PCR and Sanger sequencing. The phage sensitivity of the deletion mutant strains was then characterized via double-layer agar plate assays and adsorption assays, as described above.

### Construction of Complementation Strains

Complementation strains were also generated to determine whether wild-type copies of the deleted genes could restore phage sensitivity to the deletion mutants. *tonB*, *fhuA*, *fepA*, *ompF*, *waaG*, *tsx*, *ompA*, and *fadL* were separately amplified using the CPEC technique and cloned into pET-28a to generate complementation plasmids. The complementation plasmids, designated pET-28a-xxx, were then transformed into their corresponding deletion mutations by electroporation to obtain complemented strains. The phage sensitivity of the complementation strains was confirmed by double-layer agar plate assays and adsorption assays, as described above.

### Screening of Polyvalent Phage-Resistant Strains

Eight phages isolated in this study that infect *E. coli* BL21 (DE3) via different receptors were mixed to produce a phage cocktail. BL21 (DE3) mutant strains were then screened against the phage cocktail using the methods described above. Spotting assays and double-layer agar plate assays were used to identify the susceptibilities of the phage-resistant strains to the phage cocktail and to each of the phages individually. A polyvalent phage-resistant strain showing resistance to all the phages in the cocktail was selected for further analysis and was designated strain PR8.

### Analysis of the Polyvalent Phage-Resistant Strain

A total of 32 phages isolated from China that can infect *E. coli* BL21 (DE3) were collected in our laboratory. The phage sensitivity of strain PR8 was evaluated by a double-layer agar plate method. Bacterial growth curves were generated for the BL21 (DE3) and PR8 strains to identify any differences in bacterial counts between the two strains during propagation. Briefly, overnight cultures were used to inoculate fresh LB medium at a ratio of 1:100 (v/v). The cultures were then incubated at 37°C, 220 rpm for 12 h. Three replicate samples (1 mL each) were collected every hour to determine the OD_600_ of the cultures. Three replicates were conducted for determination.

In addition, to identify differences in protein expression between polyvalent phage-resistant strain PR8 and wild-type strain BL21 (DE3), enhanced green fluorescent protein EGFP was selected as an indicator protein. Briefly, *egfp* was cloned into pET-28a, generating recombinant plasmid pET-28a-egfp, which was then transformed into PR8 and BL21 (DE3) electrocompetent cells, respectively. Recombinant strains containing pET-28a-egfp were separately cultured overnight and then used to inoculate fresh LB medium at a ratio of 1:100 (v/v). Cultures were incubated at 37°C with shaking to OD_600_ = 0.6 before the addition of isopropyl β-D-1-thiogalactopyranoside (IPTG) to a final concentration of 0.8 mM to induce the expression of EGFP. Three parallel 200 μL samples were taken in 96-well plates every hour and the fluorescence values were determined using a Multi-Mode Microplate Detection Platform SpectraMax^®^ i3 (excitation 485 nm, emission 535 nm). A strain that did not carry the expression vector was used as the control. Three replicates were conducted for determination. Fluorescence value = experimental group fluorescence value - control group fluorescence value.

## Results

### Phage Morphology

**FIGURE 1:**
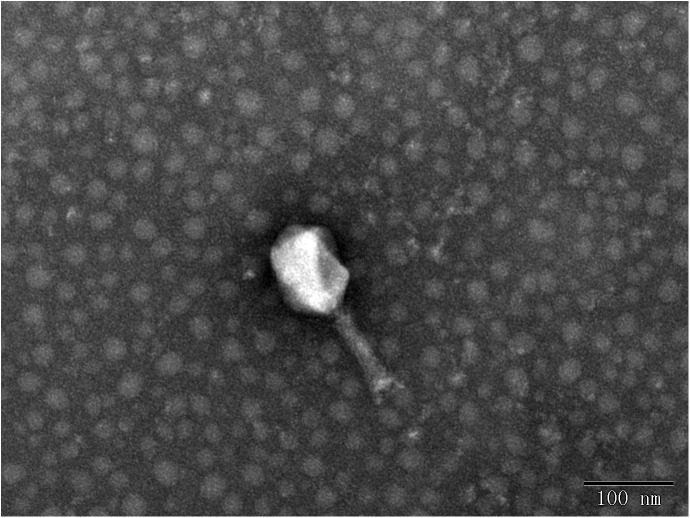
Transmission electron micrograph images of *Escherichia coli* phage vB_EcoM_IME281.

**FIGURE 2:**
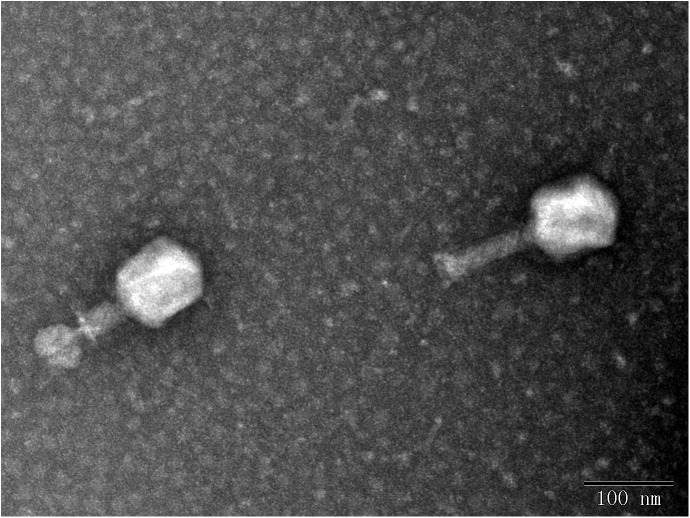
Transmission electron micrograph images of *Escherichia coli* phage vB_EcoM_IME339.

**FIGURE 3:**
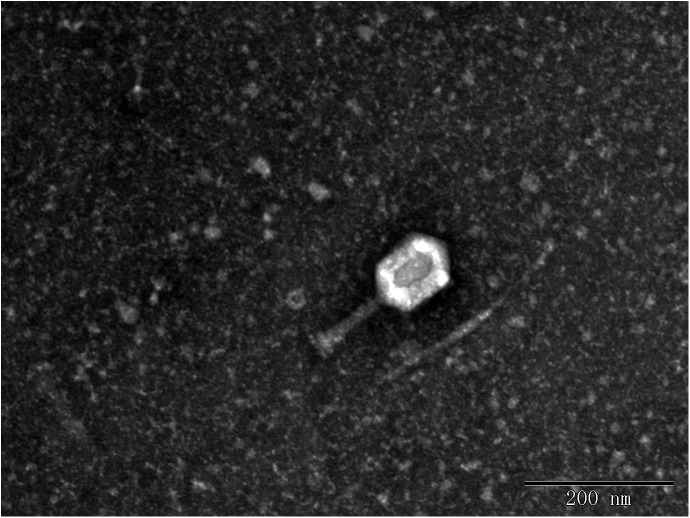
Transmission electron micrograph images of *Escherichia coli* phage vB_EcoM_IME340.

**FIGURE 4:**
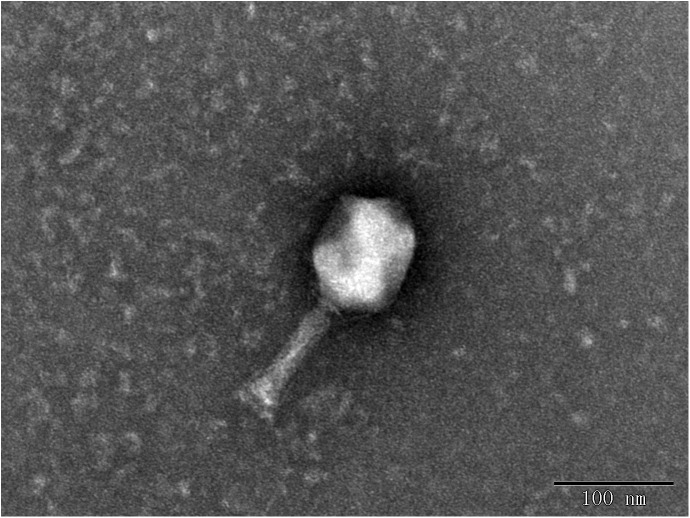
Transmission electron micrograph images of *Escherichia coli* phage vB_EcoM_IME341.

**FIGURE 5:**
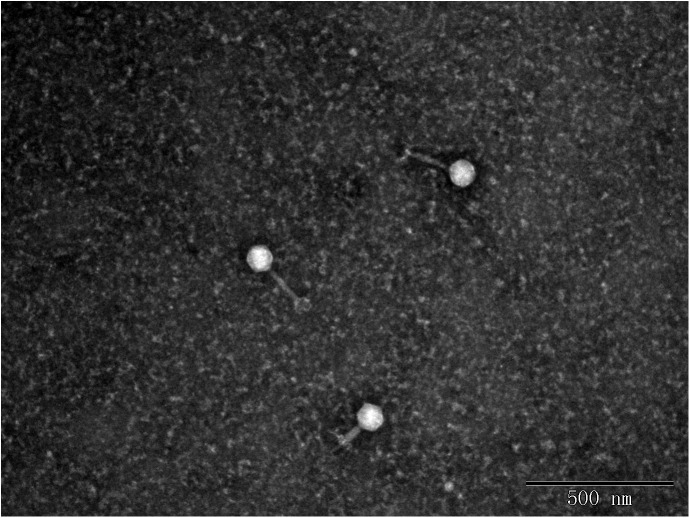
Transmission electron micrograph images of *Escherichia coli* phage vB_EcoM_IME338.

**FIGURE 6:**
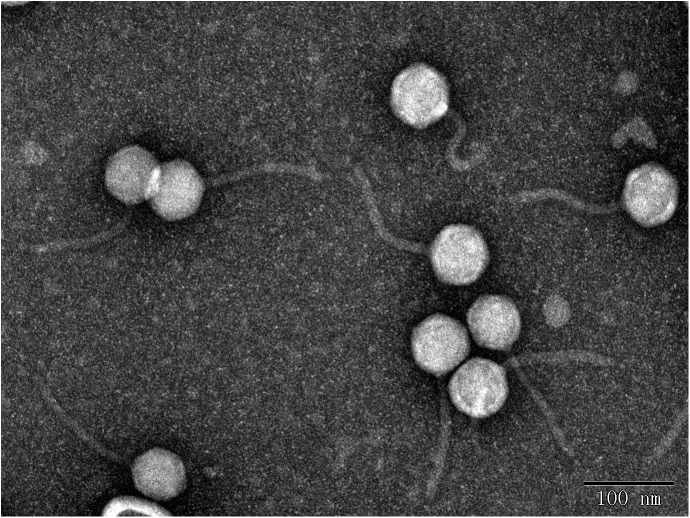
Transmission electron micrograph images of *Escherichia coli* phage vB_EcoS_IME18.

**FIGURE 7:**
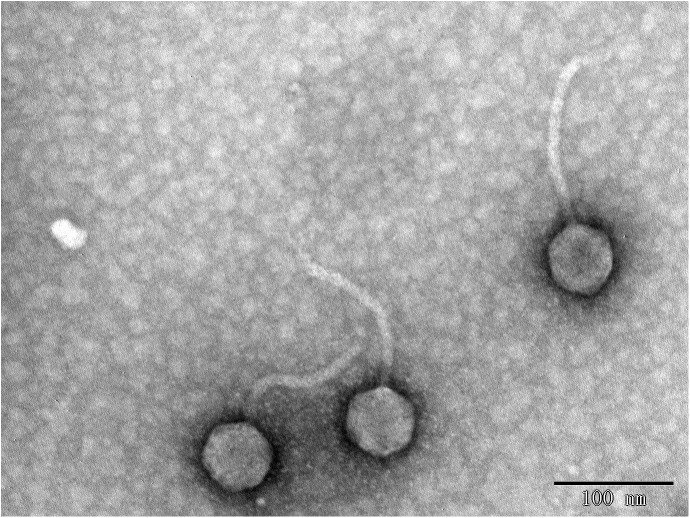
Transmission electron micrograph images of *Escherichia coli* phage vB_EcoS_IME253.

**FIGURE 8:**
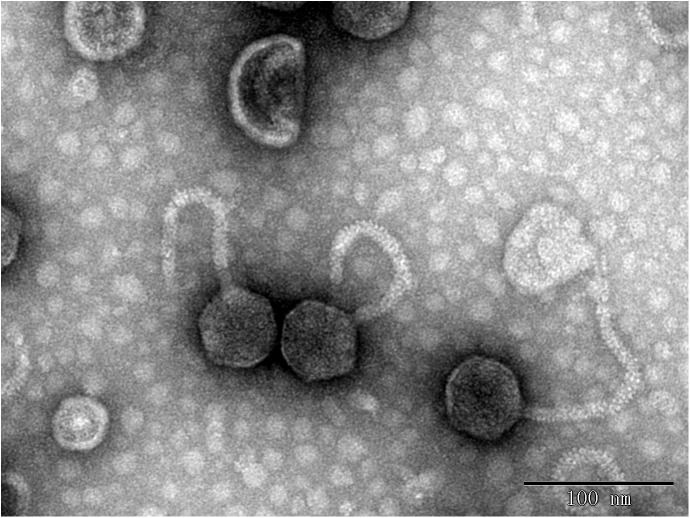
Transmission electron micrograph images of *Escherichia coli* phage vB_EcoS_IME347.

In this study, eight phages were sequentially separated from sewage (Table 2). Phages vB_EcoS_IME18 (IME18), vB_EcoS_IME253 (IME253), vB_EcoM_IME281 (IME281), vB_EcoM_IME338 (IME338), vB_EcoM_IME339 (IME339), vB_EcoM_IME340 (IME340), vB_EcoM_IME341 (IME341), and vB_EcoS_IME347 (IME347) formed clear, bright plaques on soft agar plates (Supplementary Figure S2). TEM imaging showed that phages IME281 (Figure 1), IME339 (Figure 2), IME340 (Figure 3), and IME341 (Figure 4) had a prolate head and a tail with a contractile sheath. The elongated icosahedral head was ∼100 nm long and 80 nm wide, while the tail tube was ∼100 nm long. Phage IME338 (Figure 5) had an icosahedral head approximately 60 nm wide and a tail with a contractile sheath approximately 100 nm long. Phages IME18 (Figure 6), IME253 (Figure 7) and IME347 (Figure 8) had an icosahedral head and a non-contractile tail, with a head diameter of approximately 60 nm and a tail length of approximately 160 nm.

**Table 2:** Phages isolated in this study.

Phage	Genomes size (bp)	Species	Microscopy	MOI	Burst size (PFU/cell)	Receptor
vB_EcoS_IME18	50,354	*T1virus*	*Siphoviridae*	0.01	223	FhuA
vB_EcoS_IME253	46,717	*Rtpvirus*	*Siphoviridae*	0.01	186	FepA
vB_EcoM_IME281	170,531	*Js98virus*	*Myoviridae*	0.001	153	OmpF
vB_EcoM_IME338	85,675	*Felix01virus*	*Siphoviridae*	0.001	81	LPS
vB_EcoM_IME339	164,366	*T4virus*	*Myoviridae*	0.001	91	Tsx
vB_EcoM_IME340	165,549	*T4virus*	*Myoviridae*	0.001	95	OmpA
vB_EcoM_IME341	172,379	*Js98virus*	*Myoviridae*	0.001	246	FadL
vB_EcoS_IME347	50,048	*T1virus*	*Siphoviridae*	0.01	145	YncD

### Optimal MOI and One-Step Growth Curves

**FIGURE 9:**
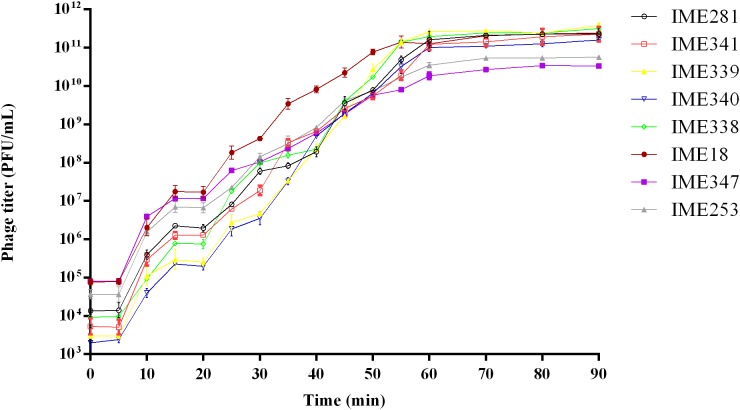
One-step growth curves of *Escherichia coli* phages vB_EcoS_IME18, vB_EcoS_IME253, vB_EcoM_IME281, vB_EcoM_IME338, vB_EcoM_IME339, vB_EcoM_IME340, vB_EcoM_IME341, and vB_EcoS_IME347.

Multiplicity of infection analysis assays showed that the optimal MOIs for phages IME18, IME253, IME281, IME338, IME339, IME340, IME341, and IME347 were 0.01, 0.01, 0.001, 0.001, 0.001, 0.001, 0.001, and 0.01, respectively. One-step growth curve analyses revealed that all of the phages had a latency period of about 5 min (Figure 9). The burst sizes of phages IME18, IME253, IME281, IME338, IME339, IME340, IME341, and IME347 were 223, 186, 153, 81, 91, 95, 246, 145 PFU/cell, respectively. The final titers of all phages exceeded 10^10^ PFU/mL, indicating that they are highly infectious toward *E. coli* BL21 (DE3).

### Phage Genome Analysis

**FIGURE 10:**
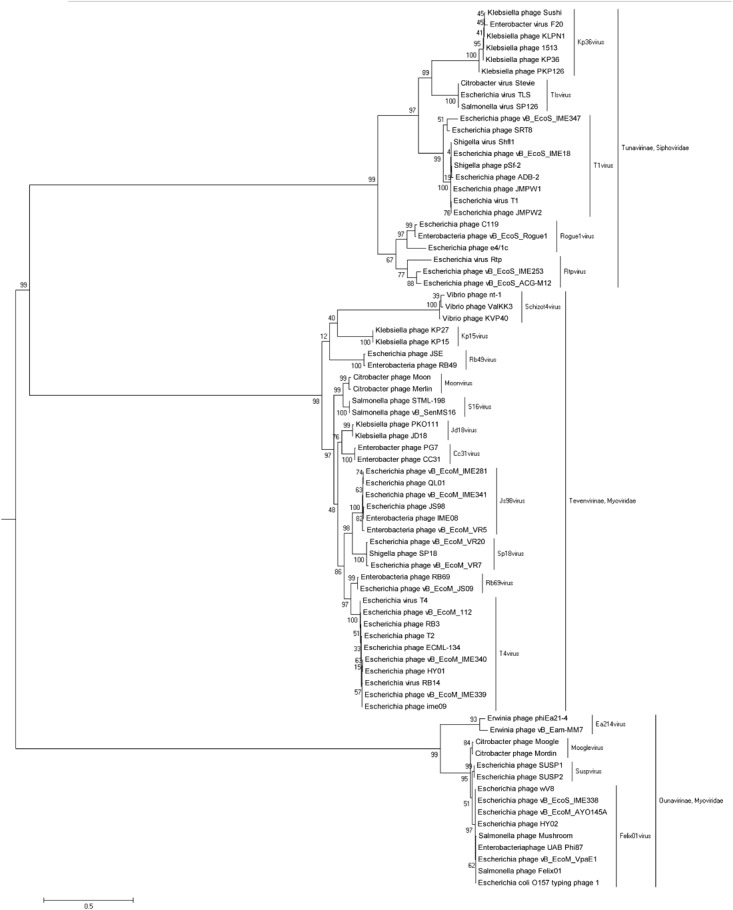
Phylogenetic tree based on the terminase large subunit protein amino acid sequences of *Escherichia coli* phages vB_EcoS_IME18, vB_EcoS_IME253, vB_EcoM_IME281, vB_EcoM_IME338, vB_EcoM_IME339, vB_EcoM_IME340, vB_EcoM_IME341, and vB_EcoS_IME347.

The genomes of phages IME18 (GenBank Accession No. MH051911), IME253 (GenBank Accession No. KX130960), IME281 (GenBank Accession No. MH051913), IME338 (GenBank Accession No. MH051914), IME339 (GenBank Accession No. MH051915), IME340 (GenBank Accession No. MH051916), IME341 (GenBank Accession No. MH051917), and IME347 (GenBank Accession No. MH051918) were 50,354, 46,717, 170,531, 85,675, 164,366, 165,549, 172,379, and 50,048 bp in size, respectively, with GC contents of 45.6, 44.2, 39.4, 38.7, 35.6, 35.5, 39.5, and 49.7%, respectively. Phylogenetic analysis based on the amino acid sequence of the large subunit of the terminase from each phage showed that IME281 and IME341 were most closely related to *Js98virus*, *Tevenvirinae*, *Myoviridae*; IME339 and IME340 were most closely related to *T4virus*, *Tevenvirinae*, *Myoviridae*; IME338 was most similar to *Felix01virus*, *Ounavirinae*, *Myoviridae*; IME18 and IME347 were most closely related to *T1virus*, *Tunavirinae*, *Siphoviridae*, and IME253 was most closely related to *Rtpvirus*, *Tunavirinae*, *Siphoviridae* (Figure 10).

### Analysis of Bacteriophage-Insensitive Mutants

Three *E. coli* mutants that were insensitive to each of the isolated phages were randomly selected for whole-genome sequencing. The mutations putatively responsible for phage resistance were then identified by whole genome comparison with the sequence of phage-sensitive strain BL21 (DE3). We searched for phage receptor-associated variations in the genomes of the BIMs, with all results listed in Supplementary Table S2. Analysis revealed that mutations within *fhuA*, *fepA*, *ompF*, *waaG*, *tsx*, *ompA*, *fadL*, and *yncD* were present in the strains showing resistance to phages IME18, IME253, IME281, IME338, IME339, IME340, IME341, and IME347, respectively. *fhuA*, *fepA*, *ompF*, *tsx*, *ompA*, *fadL*, and *yncD* all encode different bacterial outer membranes proteins, while *waaG* participates in the synthesis of LPS. These findings indicate that the identified genes play an important role in the infection of BL21 (DE3) by the corresponding bacteriophages.

### Identification and Confirmation of Phage Receptor-Related Genes

YncD is a receptor protein of phage IME347 for BL21 (DE3; Li et al., 2018). To confirm whether *fhuA*, *fepA*, *ompF*, *waaG*, *tsx*, *ompA*, and *fadL* were phage receptor-related genes with a role in the observed phage resistance, we constructed deletion mutants of each of these genes using the Scarless Cas9 Assisted Recombineering system. FhuA is the receptor for bacteriophage T1, and the infection process requires the function of TonB (Hantke and Braun, 1978). To determine whether the infection of phages IME18, IME253, and IME347 is dependent on TonB, we also constructed a *tonB* deletion mutant strain. During gene editing, the bacterial genome is cleaved by the Cas9 protein under the guidance of the sgRNA, after which the homologous recombinase integrates the homologous arm-containing oligonucleotide into the cleaved genome. PCR and Sanger sequencing confirmed that all of the target genes were successfully knocked out in the current study.

Phage receptor gene deletion mutants showed phage-resistant phenotypes in double-layer plate assays. Deletion mutant Δ*tonB* was not infected by phages IME18 and IME253 but was infected by IME347, indicating that IME18 and IME253 phage infection is TonB-dependent, whereas phage IME347 phage infection is TonB-independent. Deletion mutants Δ*fhuA*, Δ*fepA*, Δ*ompF*, Δ*waaG*, Δ*tsx*, Δ*ompA*, Δ*fadL*, and Δ*yncD* were not infected by phages IME18, IME253, IME281, IME338, IME339, IME340, IME341, and IME347, respectively, but could be infected by other isolated phages. In addition, the phage adsorption capacity of the phage receptor gene deletion mutants was significantly reduced compared with that of phage-sensitive strain BL21 (DE3; Table 3). Ddouble-layer agar plate assays showed that complementation of the mutant strains with the wild-type gene restored sensitivity to phage infection in all cases, while adsorption assays revealed that complementation also restored the adsorption capacity of the phages (Table 3). Together, these results verified that the receptors for phages IME18, IME253, IME281, IME338, IME339, IME340, IME341, and IME347 are FhuA, FepA, OmpF, LPS, Tsx, OmpA, FadL, and YncD, respectively, and that the receptors are not shared.

**Table 3:** The adsorption rate of phages.

Phage	Receptor	BL21 (DE3; PFU/cell/ mL/min)	Deletion (PFU/cell/ mL/min)	Complementation (PFU/cell/ mL/min)
vB_EcoS_IME18	FhuA	5.7047E–10	3.3557E–11	5.03356E–10
vB_EcoS_IME253	FepA	4.69231E–10	3.84615E–11	4.30769E–10
vB_EcoM_IME281	OmpF	8.69359E–10	7.60095E–11	8.50356E–10
vB_EcoM_IME338	WaaG	4.61538E–10	8.02676E–11	4.14716E–10
vB_EcoM_IME339	Tsx	9.13043E–10	1.08696E–11	8.58696E–10
vB_EcoM_IME340	OmpA	1.05516E–09	5.27578E–11	1.03118E–09
vB_EcoM_IME341	FadL	7.80105E–10	4.18848E–11	7.27749E–10
vB_EcoS_IME347	YncD	3.97394E–10	3.25733E–11	3.71336E–10

### Analysis of a Polyvalent Phage-Resistant Strain

**FIGURE 11:**
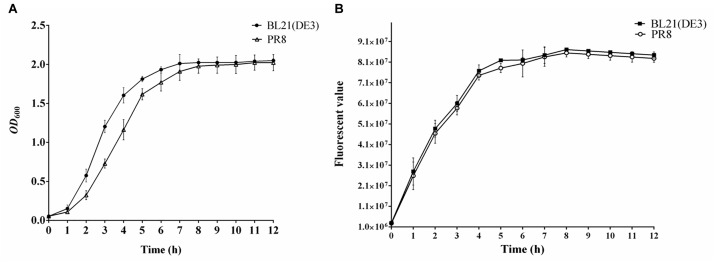
Evaluation of recombinant protein expression performance of *Escherichia coli* PR8. **(A)** Growth curve of *E. coli* BL21 (DE3) and PR8. **(B)** Fluorescence value of EGFP expressed by *E. coli* BL21 (DE3) and PR8.

Strain PR8 was obtained by screening using a cocktail of the above eight phages, twice. Whole genome sequencing of strain PR8 revealed that the strain also had mutations in genes *fhuA*, *fepA*, *ompF*, *waaG*, *tsx*, *ompA*, *fadL*, and *yncD* relative to *E. coli* BL21 (DE3; Supplementary Table S3). Double-layer agar plate assays showed that the selected polyvalent phage resistant-strain PR8 was resistant to 23 phages (Table 4). Of the 23 phages, eleven were identified as *Myoviridae* phages, seven were identified as *Siphoviridae* phages and five were identified as *Ackermannviridae* phages. The results of the BLAST analysis of phage whole genomes showed no identity between phages of different subfamilies and high identity between phages of the same genus (Supplementary Table S4). To confirm that strain PR8 was also capable of high-level recombinant protein expression, growth curve and EGFP protein expression analyses were conducted. The results showed that polyvalent phage-resistant strain PR8 had a slightly lower OD_600_ than the wild-type strain during the first 8 h of incubation, but that the cell density increased to wild-type levels at 8–12 h post-inoculation (Figure 11A). The strains PR8 and BL21 (DE3) grew to OD_600_ = 0.6 and expressed recombinant EGFP in the same conditions. Fluorescence analysis showed that there was only a small difference in fluorescence between the two strains over 8h of expression (Figure 11B).

**Table 4:** Phages to which *Escherichia coli* PR8 was resistant.

Phage	Description	Source
vB_EcoM_IME339	*Myoviridae*, *Tevenvirinae*, *T4virus*	Beijing
vB_EcoM_IME340	*Myoviridae*, *Tevenvirinae*, *T4virus*	Beijing
IME391	*Myoviridae*, *Tevenvirinae*, *T4virus*	Qingdao
vB_EcoM_IME281	*Myoviridae*, *Tevenvirinae*, *Js98virus*	Beijing
vB_EcoM_IME341	*Myoviridae*, *Tevenvirinae*, *Js98virus*	Beijing
IME412	*Myoviridae*, *Tevenvirinae*, *Js98virus*	Henan
IME361	*Myoviridae*, *Tevenvirinae*, *Rb69virus*	Qingdao
IME362	*Myoviridae*, *Tevenvirinae*, *Rb69virus*	Qingdao
vB_EcoM_IME338	*Myoviridae*, *Ounavirina*, *Felix01virus*	Beijing
IME364	*Myoviridae*, *Ounavirina*, *Felix01virus*	Wuhan
IME365	*Myoviridae*, *Ounavirina*, *Felix01virus*	Wuhan
T1	*Siphoviridae, Tunavirinae, T1virus*	–
vB_EcoS_IME18	*Siphoviridae*, *Tunavirinae*, *T1virus*	Beijing
vB_EcoS_IME167	*Siphoviridae*, *Tunavirinae*, *T1virus*	Beijing
vB_EcoS_IME347	*Siphoviridae*, *Tunavirinae*, *T1virus*	Beijing
JMPW1	*Siphoviridae*, *Tunavirinae*, *T1virus* (Shen et al., 2016)	–
vB_EcoS_IME253	*Siphoviridae*, *Tunavirinae*, *Rtpvirus*	Beijing
SSL-2009a	*Siphoviridae*, *HK578likevirus* (Li et al., 2019)	–
IME360	*Ackermannviridae*, *Cvivirinae*, *Cba120virus*	Wuhan
IME366	*Ackermannviridae*, *Cvivirinae*, *Cba120virus*	Wuhan
IME371	*Ackermannviridae*, *Cvivirinae*, *Cba120virus*	Wuhan
IME375	*Ackermannviridae*, *Cvivirinae*, *Cba120virus*	Wuhan
IME377	*Ackermannviridae*, *Cvivirinae*, *Cba120virus*	Wuhan

## Discussion

Two *T4virus* phages isolated in this study used OMPs as receptors to infect *E. coli* BL21 (DE3). The receptor for phage IME339 is Tsx, which serves as a substrate-specific channel for nucleosides and deoxynucleosides. Structures of Tsx with bound nucleosides show that there are at least three distinct binding sites in the channel (Nuc0, Nuc1, and Nuc2). Mutations Phe27Leu, Gly28Arg (Glu), Ser217Arg, Gly239Asp, and Gly240Asp result in a Tsx protein defective in nucleoside transport (Fsihi et al., 1993; Ye and van den Berg, 2004). The amino acid mutation sites of the phage IME339-resistant strain and PR8 were Leu149Glu, Phe18fs, Met1_Ter295del, and Trp171Ter (where “fs” indicates a frame shift). Residual Leu149 is extracellular, close to loop 4. Mutation Leu149Glu affected the infection of phage IME339. In addition, residues 198–207 of *E. coli* K12 Tsx might be part of the Tsx-specific phage (T6, T6h3.1,Ox1, H1, H3, H8, H9, K18) receptor region, and substitutions Asn249Lys and Asn254Lys (Tyr) strongly impaired the phage T6 receptor function of Tsx (Krieger-Brauer and Braun, 1980; Schneider et al., 1993; Nieweg and Bremer, 1997). The receptor for phage IME340 is OmpA, which is one of the major OMPs in *E. coli*, with 100,000 copies typically found per cell (Koebnik et al., 2000; Ortiz-Suarez et al., 2016). OmpA has multiple functions. For example, OmpA can act as a receptor for colicin K and colicin L, a pore protein that allows slow penetration of small solutes, the adhesin/invasin to produce pathogenicity, involved in biofilm formation, and participates in innate immunity system (Smith et al., 2010). In *E. coli* K-12, OmpA serves as a receptor for many T-even-like phages, with four mutational alterations (residue 25 in loop 1, residue 70 in loop 2, residue 110 in loop 3, and residue 154 in loop 4) found to affect the ability of OmpA to function as a phage receptor (Morona and Henning, 1984; Morona et al., 1984; Nieweg and Bremer, 1997). OmpA can also act as a host-specific factor in *Shigella* species that mediates phage Sf6 (*P22virus* subfamily, *Podoviridae* family) binding, with loops 2 and 4 being the most critical (Parent et al., 2014; Porcek and Parent, 2015). In this study, the amino acid mutations in OmpA in phage IME340-resistant strain and PR8 were Val122fs, Gln38Ter, Met1_Ter347del, and Lys33Ter. The site of phage IME340 is located between residues 122 and 347.

Two *Js98virus* phages isolated in this study also used OMPs as receptors to infect *E. coli* BL21 (DE3). The receptor for phage IME281 is the osmotically regulated cation-selective OmpF protein, which consists of three monomeric channels (Cowan et al., 1992). OmpF can be used as a receptor for colicin *N*, a causative agent and an antibiotic channel. OmpF is a receptor protein for phage K20, and substitution of residues exposed on the surfaces of loops 5, 6, or 7 prevents the binding of K20 without affecting the channel activity of OmpF (Silverman and Benson, 1987; Traurig and Misra, 2010). In addition, infection by *Yersinia* phages TG1 (*Tg1virus* genus, *Myoviridae* family) and ΦR1-RT (*Tg1virus* genus, *Myoviridae* family) is dependent on temperature-regulated expression of OmpF (Leonvelarde et al., 2016). The amino acid mutation sites in OmpF of the phage IME281-resistant strain and PR8 were Tyr79_Val128del, Asp76fs, Met1_Ter363del, and Thr77_Tyr128del. Tyr79_Val128del results in a deletion of loop 3 demonstrating that loop 3 has phage receptor function. The receptor protein for phage IME341 was identified as a monomer of FadL, which is required for the transport of long-chain fatty acids through the outer membrane and also participates in the uptake of hydrophobic compounds, including aromatic hydrocarbons, for biodegradation (van den Berg et al., 2004). Residues Phe448, Pro428, Val410, and Ser397 are required for optimal levels of long-chain fatty acid transport and that amino acid residues Pro428 and Val410 are essential for long-chain fatty acid binding (Kumar and Black, 1993). FadL also acts as a receptor protein for phage T2, and its exposed extracellular loop (residues 28–160.) is required for phage T2 binding (Cristalli et al., 2000). The mutations of FadL in the phage IME341-resistant strain and PR8 were Asp34Ter, Leu161Val, Leu394Glu, and Met1_Ter447del. Residues Leu161 and Leu394 in FadL are extracellular. Mutations Leu161Val and Leu394Glu seriously affected the infection of phage IME341.

Infection of *E. coli* BL21 (DE3) by phages IME18 and IME253 was TonB-dependent, with FhuA and FepA used as receptors, respectively. The FhuA amino acid mutation sites of the phage IME18-resistant strain and PR8 were Ser675_Trp704del, Thr629fs, Phe519fs, and Met416_Arg417del. Residues Ser675_Trp704 and Met416_Arg417 are related to receptor function of phage IME18. In the phage IME253-resistant strain and PR8, *fepA* was deleted. FhuA and FepA belong to the family of TonB-dependent receptors. FhuA is mainly involved in the binding and absorption of ferrichrome and colicin M, and is a receptor for *Siphoviridae* bacteriophages T1, T5, phi80, and UC-1 (Killmann et al., 1995; Endriss and Braun, 2004). FepA is mainly involved in the transport of ferric enterobactin and is a receptor for T5-like phage H8 (Rabsch et al., 2007). Introduction of a foreign peptide after FepA residues 55, 142, or 324 can severely impair receptor function for ferric enterobactin, colicin D and colicin B. However, the introduction of a foreign peptide after residues 204 or 635 only restricts FepA’s function for colicin B and colicin D (Armstrong et al., 1990). In addition, TonB-dependent receptor BtuB, which is required for the binding and transport of vitamin B12, is a receptor for T5-like phages EPS7 and SPC35 (Hong et al., 2008; Kim and Ryu, 2011).

Lipopolysaccharides is an important component of the outer membrane of Gram-negative bacteria and consists of three parts: lipid A, core oligosaccharide, and O-antigen. The complete LPS structure is called smooth (S) type LPS, while LPS lacking the O-antigen is referred to as rough (R) type. Lipid A is located on the innermost side of LPS and is usually conserved, while the polysaccharide polymer composed of O-antigen, the structural composition of which is highly variable, can extend to the outside of the cell membrane. Generally, the host range of phages capable of cleaving S-type strains is broader than that of phages targeting R-type cells (Rakhuba et al., 2010). *waaG* is involved in the synthesis of LPS in *E. coli*, and encodes a glycosyltransferase responsible for transferring and linking the primary glucose residue of the outer core of the LPS core oligosaccharide to the inner core of the LPS (Heinrichs et al., 2010). Deletion of this gene results in the loss of the O-antigen and the outer core of the core oligosaccharide of LPS. Felix01 phage has been reported to use LPS as a receptor, and here we demonstrate that *Felix01virus* phage IME338 also uses LPS as a receptor (Hudson et al., 1978).

In this study, we identified two *T4virus* phages (IME339 and IME340), two *Js98virus* phages (IME281 and IME341), one *Felix01virus* phage (IME338), two *T1virus* phages (IME18 and IME347), and one *Rtpvirus* phage (IME253), all of which used different receptors for infection of *E. coli* BL21 (DE3). We confirmed that the receptors for phages IME18, IME253, IME281, IME338, IME339, IME340, IME341, and IME347 are FhuA, FepA, OmpF, LPS, Tsx, OmpA, FadL, and YncD, respectively, and that none of the receptors are shared. We then identified a polyvalent phage-resistant BL21 (DE3) mutant strain, designated PR8, using a screening assay based on a phage cocktail consisting of the eight identified phages; PR8 is resistant to 23 tested phages. Strain PR8 not only resists infection by multiple phages but also has the ability to express high levels of recombinant protein, indicating that it is likely to be a valuable strain for production of recombinant protein. However, the mechanisms of interactions between phages and their hosts are not fully understood, and further research is needed to provide a theoretical basis for phage contamination control. Replacement of all UAG termination codons in *E. coli* with UAA enhances host resistance to T7 phage (Lajoie et al., 2013). Therefore, even greater codon changes may allow a host to completely avoid phage infection (Ostrov et al., 2016). In the near future, it will be possible to produce synthetic cells that are protected from phage infection. The results of the current study provide important information for such endeavors.

## Author Contributions

YT, JW, and PL conceived and designed the experiments. PL carried out the experiments and wrote the manuscript. All authors analyzed the data, read, and approved the final manuscript.

## Conflict of Interest Statement

The authors declare that the research was conducted in the absence of any commercial or financial relationships that could be construed as a potential conflict of interest.

## Footnotes

1 http://rast.nmpdr.org

2 https://www.dna20.com/products/crispr
