# Supplementary material for: Screening of Polyvalent Phage-Resistant Escherichia coli Strains Based on Phage Receptor Analysis
Source: Front Microbiol. 2019 Apr 18;10:850. doi: 10.3389/fmicb.2019.00850 (PMC6499177; doi:10.3389/fmicb.2019.00850)
Supplement: Supplementary file 1 [file Data_Sheet_1.pdf]

Supplementary Material

Screening of Polyvalent Phage-Resistant Escherichia coli Strains Based on Phage Receptor Analysis

Ping Li[1,2], Hong Lin[1], Zhiqiang Mi[2], Shaozhen Xing[2], Yigang Tong[2,3*], Jingxue Wang[1*]

*** Correspondence:** Jingxue Wang: snow@ouc.edu.cn; Yigang Tong: tong.yigang@gmail.com

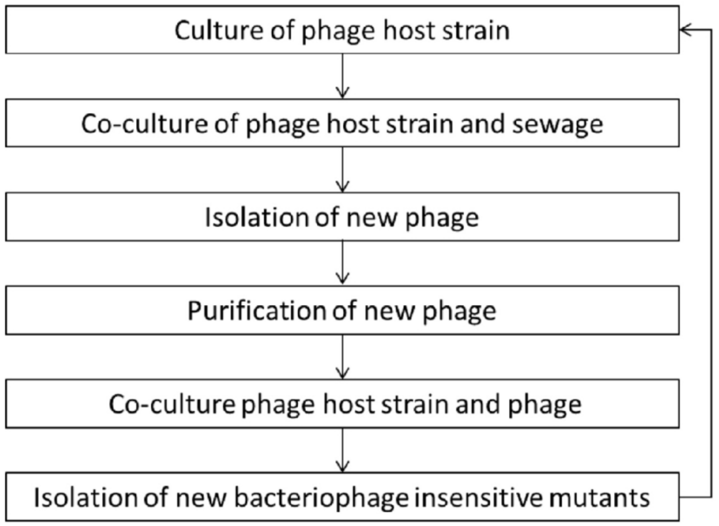

Figure S1 Method for isolating bacteriophage insensitive mutants.

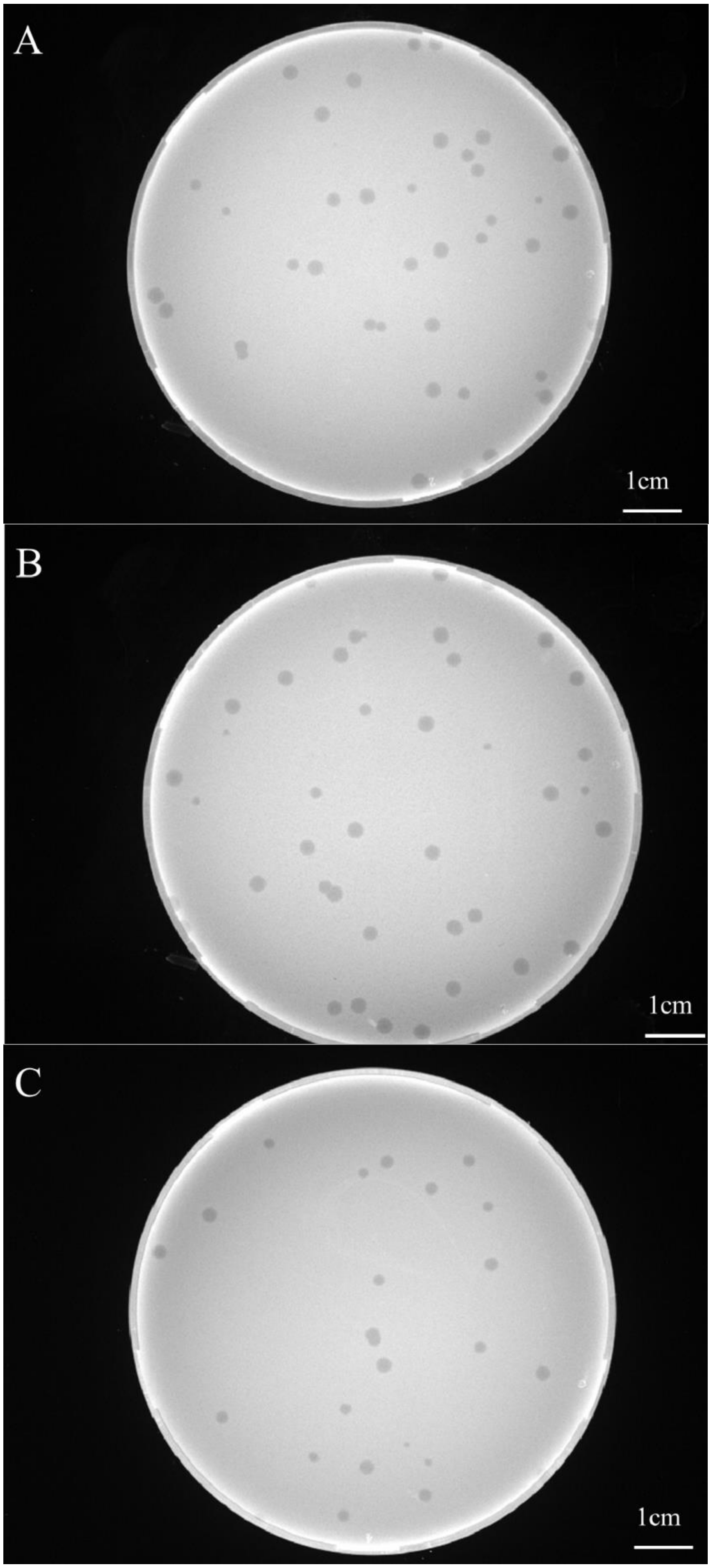
A
1cm
B
1cm
C
1cm

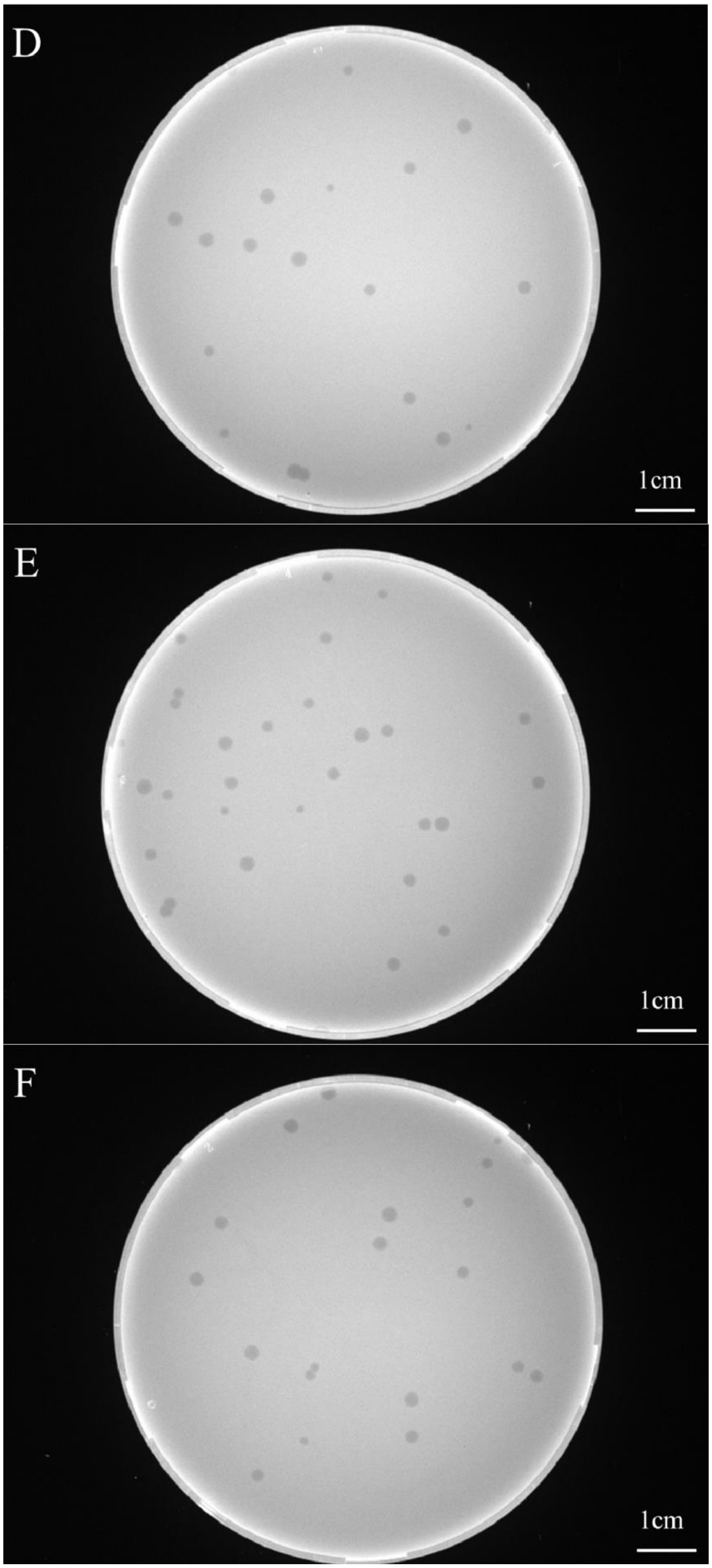
D
1cm
E
1cm
F
1cm

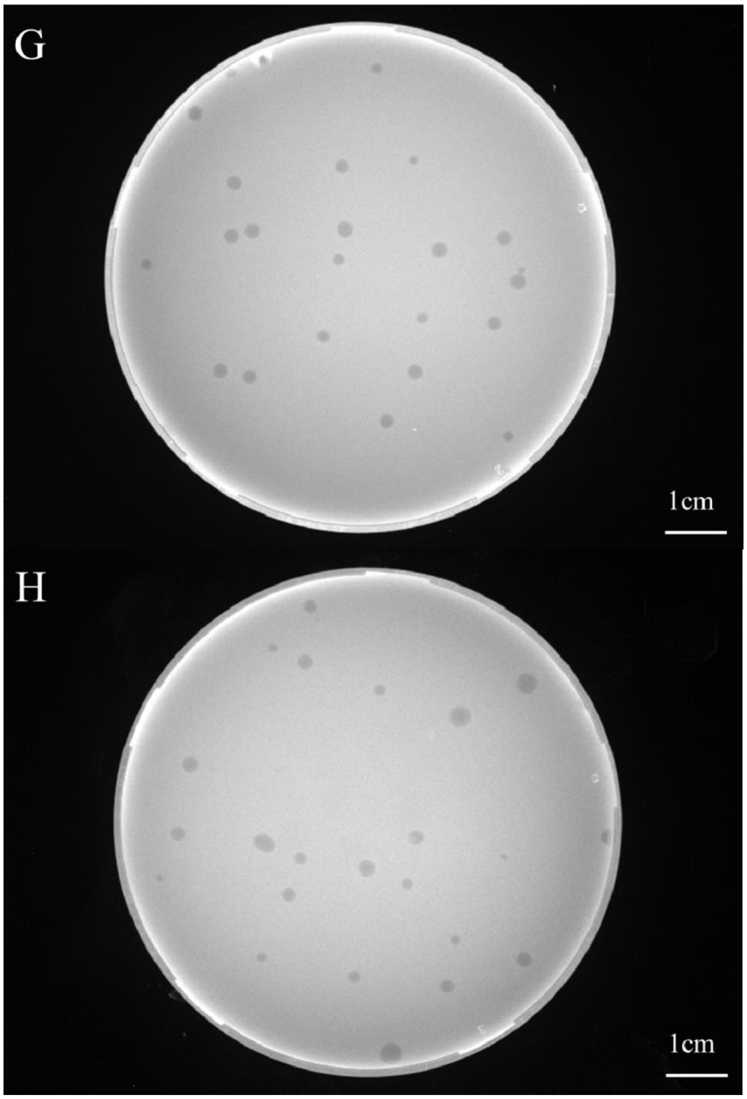

Figure S2 Plaque morphology of phages vB_EcoS_IME18 (Figure S2 (A)), vB_EcoS_IME253 (Figure S2 (B)), vB_EcoM_IME281 (Figure S2 (C)), vB_EcoM_IME338 (Figure S2 (D)), vB_EcoM_IME339 (Figure S2 (E)), vB_EcoM_IME340 (Figure S2 (F)), vB_EcoM_IME341 (Figure S2 (G)), and vB_EcoS_IME347 (Figure S2 (H)).

Table S1 Primers used in this study.

Primer	Base sequence (5′→ 3′)	Description
pKDsg-tonB-F	GTGCTTCTTTTGGCGGTTCGGTTTTAGAGCTAGAA ATAGCAAG	To construct counter-selection plasmid pKDsg-tonB.
pKDsg-tonB-R	CGAACCGCCAAAAGAAGCACGTGCTCAGTATCTC TATCACTGA	To construct counter-selection plasmid pKDsg-tonB.
pKDsg-fhuA-F	ATCACGTTTATCGGTCGTCCGTTTTAGAGCTAGA AATAGCAAG	To construct counter-selection plasmid pKDsg-fhuA.
pKDsg-fhuA-R	GGACGACCGATAAACGTGATGTGCTCAGTATCTC TATCACTGA	To construct counter-selection plasmid pKDsg-fhuA.
pKDsg-fepA-F	TGCCAAATCGCCCGTGGTCGTTTTAGAGCTAGAA ATAGCAAG	To construct counter-selection plasmid pKDsg-fepA.
pKDsg-fepA-R	GACCACGGGCGATTTGGCAGTGCTCAGTATCTCT ATCACTGA	To construct counter-selection plasmid pKDsg-fepA.
pKDsg-ompF-F	GACTTCTTCGTTGGTCGTGTGTTTTAGAGCTAGAA ATAGCAAG	To construct counter-selection plasmid pKDsg-ompF.
pKDsg-ompF-R	ACACGACCAACGAAGAAGTCGTGCTCAGTATCTC TATCACTGA	To construct counter-selection plasmid pKDsg-ompF.
pKDsg-waaG-F	CTCCGCATTGCGCCCGTGATGTTTTAGAGCTAGA AATAGCAAG	To construct counter-selection plasmid pKDsg-waaG.
pKDsg-waaG-R	ATCACGGGCGCAATGCGGAGGTGCTCAGTATCTC TATCACTGA	To construct counter-selection plasmid pKDsg-waaG.
pKDsg-tsx-F	AACTTCAACGTTCGCTCTACCGTTTTAGAGCTAG AAATAGCAAG	To construct counter-selection plasmid pKDsg-tsx.
pKDsg-tsx-R	GGTAGAGCGAACGTTGAAGTTGTGCTCAGTATCT CTATCACTGA	To construct counter-selection plasmid pKDsg-tsx.
pKDsg-ompA-F	TGTTTCCTACCGTTTCGGTCGTTTTAGAGCTAGAA ATAGCAAG	To construct counter-selection plasmid pKDsg-ompA.
pKDsg-ompA-R	GACCGAAACGGTAGGAAACAGTGCTCAGTATCTC TATCACTGA	To construct counter-selection plasmid pKDsg-ompA.
pKDsg-fadL-F	GTCTGCTCTCGCAGTCGCAGGTTTTAGAGCTAGA	To construct counter-selection

	AATAGCAAG	plasmid pKDsg-fadL.
pKDsg-fadL-R	CTGCGACTGCGAGAGCAGACGTGCTCAGTATCTC TATCACTGA	To construct counter-selection plasmid pKDsg-fadL.
gamF	AGCATTCACTAACCCCCTTTCC	To construct counter-selection plasmid pKDsg-xxx.
gamR	AGCTGCTGGTAGTGACGCGCCC	To construct counter-selection plasmid pKDsg-xxx.
Oligo-tonB	GCA TTT AAA ATC GAG ACC TGG TTT TTC TAC TGA AAT GAT TAT GAC TTC AGC ATA AAG TCA AAA GCC TCC GAC CGG AGG CTT TTG ACT	Oligonucleotide for Δ*tonB* recombination.
Oligo-fhuA	CCC GCC AGC GTT TCG AAT ATT ATC TTA TCT TTA TAA TAA TCA TTC CTC TTT TGG GGC ACG GAT TTC CGT GCC CAT TTC ACA AGT TGG CTG	Oligonucleotide for Δ*fhuA* recombination.
Oligo-fepA	CGG GAT GCG TCG TGT TGA TGA CGA CCA TGC CCG ACA GTT GCA ATT TAA CGT CAG ATT GTT GAC AAA GTG CGC GTC GTT CAT GCC GGA TGC	Oligonucleotide for Δ*fepA* recombination.
Oligo-ompF	CGG CAG TGG CAG GTG TCA TAA AAA AAA CCA TGA GGG TAA TAA ATA TAG CAC ACC TCT TTG TTA AAT GCC GAA AAA ACA GGA CTT TGG TCC	Oligonucleotide for Δ*ompF* recombination.
Oligo-tsx	CGC CTT TTT CAC TCC CGC AAG GGA TTT TCA AAC AGT GGC ATA CAT TTA TGA AAA TGC CGG GAT TTA TTC CCG GCA TTT CTG ATT GTT AAC	Oligonucleotide for Δ*tsx* recombination.
Oligo-ompA	TGG AGA TAT TCA TGG CGT ATT TTG GAT GAT AAC GAG GCG CAA AAA GTT CTC GTC TGG TAG AAA AAC CCC GCT GCT GCG GGG TTT TTT TTG	Oligonucleotide for Δ*ompA* recombination.
Oligo-fadL	CAC GTA ACA TAG TTT GTA TAA AAA TAA ATC ATT GAG GTT ATG GTC TAA CAC GTT CGC CTG GAT AAA GTC ACC TGC ATA GCA GGT GAC TTT	Oligonucleotide for Δ*fadL* recombination.
Oligo-waaG	TGC TGT CGA TAA ATT ACT GCC CTC CTC CAC GAC AGG TAC GTC GTT ATG GTT GAA CTT AAA GAG CCG TTT GCC ACG TTA TGG CGC GGT AAA	Oligonucleotide for Δ*waaG* recombination.
tonB-F	ATGACCCTTGATTTACCTCG	Amplification of *tonB.*
tonB-R	TTACTGAATTTCGGTGGTGC	Amplification of *tonB.*
fhuA-F	ATGGCGCGTTCCAAAACTGC	Amplification of *fhuA.*

fhuA-R	TTAGAAACGGAAGGTTGCGG	Amplification of *fhuA*.
fepA-F	ATGAACAAGAAGATTCATTC	Amplification of *fepA*.
fepA-R	TCAGAAGTGGGTGTTTACGC	Amplification of *fepA*.
ompF-F	ATGATGAAGCGCAATATTC	Amplification of *ompF*.
ompF-R	TTAGAACTGGTAAACGATA	Amplification of *ompF*.
waaG-F	ATGATCGTTGCTTTTTGTTT	Amplification of *waaG*.
waaG-R	TCAACCATCCAGACCACCCG	Amplification of *waaG*.
ompA-F	ATGAAAAAGACAGCTATCG	Amplification of *ompA*.
ompA-R	TTAAGCCTGCGGCTGAGTT	Amplification of *ompA*.
tsx-F	ATGAAAAAAACATTACTGG	Amplification of *tsx*.
tsx-R	TCAGAAGTTGTAACCTACT	Amplification of *tsx*.
fadL-F	ATGAGCCAGAAAACCCTGT	Amplification of *fadL*.
fadL-R	TCAGAACGCGTAGTTAAAG	Amplification of *fadL*.
tonB-CF	AGCCATATGGCTAGCATGACTATGACCCTTGATT TACCTCG	Construction of complementary plasmid pET-28a-tonB.
tonB-CR	TCAGCTTCCTTTCGGGCTTTGTTACTGAATTTCGG TGGTG	Construction of complementary plasmid pET-28a-tonB.
fhuA-CF	AGCCATATGGCTAGCATGACTATGGCGCGTTCCA AAACTGC	Construction of complementary plasmid pET-28a-fhuA.
fhuA-CR	TCAGCTTCCTTTCGGGCTTTGTTAGAACTGGTAAA CGATAC	Construction of complementary plasmid pET-28a-fhuA.
fepA-CF	AGCCATATGGCTAGCATGACTATGAACAAGAAG ATTCATTC	Construction of complementary plasmid pET-28a-fepA.
fepA-CR	TCAGCTTCCTTTCGGGCTTTGTCAGAAGTGGGTGT TTACGC	Construction of complementary plasmid pET-28a-fepA.
ompF-CF	AGCCATATGGCTAGCATGACTATGATGAAGCGCA	Construction of complementary

	ATATTCT	plasmid pET-28a-ompF.
ompF-CR	TCAGCTTCCTTTCGGGCTTTGTTAGAAACGGAAG GTTGCGG	Construction of complementary plasmid pET-28a-ompF.
tsx-CF	AGCCATATGGCTAGCATGACTATGAAAAAAACAT TACTGG	Construction of complementary plasmid pET-28a-tsx.
tsx-CR	TCAGCTTCCTTTCGGGCTTTGTCAGAAGTTGTAAC CTACT	Construction of complementary plasmid pET-28a-tsx.
ompA-CF	AGCCATATGGCTAGCATGACTATGAAAAAGACA GCTATCG	Construction of complementary plasmid pET-28a-ompA.
ompA-CR	TCAGCTTCCTTTCGGGCTTTGTTAAGCCTGCGGCT GAGTT	Construction of complementary plasmid pET-28a-ompA.
fadL-CF	AGCCATATGGCTAGCATGACTATGAGCCAGAAAA CCCTGT	Construction of complementary plasmid pET-28a-fadL.
fadL-CR	TCAGCTTCCTTTCGGGCTTTGTCAGAACGCGTAGT TAAAGT	Construction of complementary plasmid pET-28a-fadL.
egfp-F	AGTCATGCTAGCCATATGGCT ATGGTGAGCAAGGGCGAG	Construction of recombinant plasmid pET-28a-egfp.
egfp-R	CAAAGCCCGAAAGGAAGCTGA TGGACGAGCTGTACAAGTAA	Construction of recombinant plasmid pET-28a-egfp.
pET-F	AGTCATGCTAGCCATATGGCT	Construction of complementary plasmid pET-28a-xxx.
pET-R	CAAAGCCCGAAAGGAAGCTGA	Construction of complementary plasmid pET-28a-xxx.

Table S2 Gene variations present in bacteriophage−insensitive mutants.

Bacteriophage insensitive mutants	Reference position change	Annotations	Coding region change	Amino acid change
18-R1	172350_172439del	*fhuA*	2024_2112del	Ser675_Trp704del
18-R2	170327_172570del	*fhuA*	1886_1907del	Thr629fs
18-R3	171881_171882insA	*fhuA*	1554_1555ins	Phe519fs
253-R1	570740_572980del	*fepA*	1_2241del	Met1_Ter747del
253-R2	570740_572980del	*fepA*	1_2241del	Met1_ Ter 747del
253-R3	570740_572980del	*fepA*	1_2241del	Met1_ Ter 747del
281-R1	991689_991835del	*ompF*	238_384del	Tyr79_Val128del
281-R2	991689_991845del	*ompF*	238_374del	Asp76fs
281-R3	990984_992072del	*ompF*	1_1089del	Met1_ Ter363del
338-R1	3673550_3673567del	*waaG*	296_313del	Ala99_Ala104del
338-R2	3673567T>G	*waaG*	236A>C	Ala235Pro
338-R3	3673159	*waaG*	704G>T	Gly235Val
339-R1	397362insT	*tsx*	53_54 ins	Phe18fs
339-R2	396969A>T	*tsx*	446T>A	Leu149Glu
339-R3	3976530_397414del	*tsx*	1_885del	Met1_ Ter295del
340-R1	1024774_ 1024778del	*ompA*	365_369del	Val122fs
340-R2	1024102_ 1025142del	*ompA*	1_1041del	Met1_Ter347del
340-R3	1025031	*ompA*	112C>T	Gln38Ter
341-R1	2349673_ 2349971del	*fadL*	1043_1341del	Asp34Ter

341-R2	2349112T>G	*fadL*	482T>G	Leu161Val
341-R3	2349811T>A	*fadL*	1181T>A	Leu394Glu
347-R1	1475026_1477128del	*yncD*	1_2103del	Met1_Ter701del
347-R2	1475026_1477128del	*yncD*	1_2103del	Met1_Ter701del
347-R3	1475026_1477128del	*yncD*	1_2103del	Met1_Ter701del

Table S3 Gene variations present in *E. coli* strain PR8.

Reference position	Annotations	Coding region change	Amino acid change
171565_171570del	*fhuA*	1239_1244delTATGCG	Met416_Arg417del
3673567T>G	*waaG*	236A>C	Ala235Pro
991689_991841del	*ompF*	232_384del	Thr77_Tyr128del
1025046T>A	*ompA*	97A>T	Lys33Ter
2348631_2349971del	*fadL*	1_1341del	Met1_Ter447del
396902C>T	*tsx*	513G>A	Trp171Ter
1476453_127656del	*yncD*	673_676delCTGG	Leu225fs
4307613C>G	*hflX*	422C>G	Thr141Ser
568322_568474del	*hokE*	1_153del	Met1_Ter51del
569945_570574del	*entD*	1_630del	Met1_Ter210del
570740_572980del	*fepA*	1_2241del	Met1_Ter747del
570740_572980del	*fes*	1_1230del	Met1_Ter410del
574429_574647del	*ybdZ*	1_219del	Met1_Ter73del
574644_578525del	*entF*	1_3882del	Met1_Ter1294del
578741_579874del	*fepE*	1_1134del	Met1_Ter377del
579871_580686del	*fepC*	1_816del	Met1_Ter272del
580683_581675del	*fepG*	1_993del	Met1_Ter331del
581672_582676del	*fepD*	1_1005del	Met1_Ter335del

Table S4 The identity of phages to which *E. coli* strain PR8 was resistant.

***Myoviridae, Tevenvirinae* phages**	IME339 (query cover, ident)	IME340 (query cover, ident)	IME341 (query cover, ident)	IME391 (query cover, ident)	IME361 (query cover, ident)	IME362 (query cover, ident)	IME412 (query cover, ident)
IME281	15%, 79.57%	14%, 81.08%	90%, 93.99%	17%, 77.35%	20%, 84.76%	16%, 80.87%	87%, 95.74%
IME339		92%, 96.86%	18%, 81.48%	89%, 96.81%	37%, 79.58%	46%, 79.68%	16%, 79.77%
IME340			14%, 81.78%	91%, 97.45%	45%, 79.91%	49%, 80%	13%, 81.01%
IME341				17%, 81.47%	16%, 80.46%	14%, 81.29%	89%, 96.38%
IME391					41%, 75.18%	44%, 75.35%	16%, 81.48%
IME361						90%, 97.32%	18%, 74.91%
IME362							13%, 80.17%
***Siphoviridae, Tunavirinae,* phages**	JMPW1 (query cover, ident)	IME18 (query cover, ident)	IME167 (query cover, ident)	IME253 (query cover, ident)	IME347 (query cover, ident)		
T1	88%, 95.76%	89%, 93.00%	89%, 94.96%	2%, 86.80%	13%, 74.98%		
JMPW1		97%, 93.79%	100%, 99.94%	0%, 96.92%	10%, 78.36%		

IME18			96%,<br>92.51%	0%,<br>96.92%	0%,<br>77.81%
IME167				0%,<br>96.92%	10%,<br>78.32%
IME253					0%,<br>97.01%
Ackermannviridae, Cvivirinae, **phages**	IME366<br>(query cover, ident)	IME371<br>(query cover, ident)	IME375<br>(query cover, ident)	IME377<br>(query cover, ident)	
IME360	3%,<br>80.69%	0%,<br>0%,	0%,<br>0%,	0%,<br>80.69%	
IME366		91%,<br>98.72%	92%,<br>99.24%	97%,<br>99.33%	
IME371			91%,<br>98.47%	90%,<br>98.27%	
IME375				93%,<br>98.23%	
Myoviridae, Ounavirina, **phages**	IME364<br>(query cover, ident)	IME365<br>(query cover, ident)			
IME338	89%,<br>94.44%	88%,<br>94.49%			
IME364		97%,<br>97.49%			
